# Seroprevalence of Trichodysplasia Spinulosa–associated Polyomavirus

**DOI:** 10.3201/eid1708.110114

**Published:** 2011-08

**Authors:** Els van der Meijden, Siamaque Kazem, Manda M. Burgers, Rene Janssens, Jan Nico Bouwes Bavinck, Hester de Melker, Mariet C.W. Feltkamp

**Affiliations:** Author affiliations: Leiden University Medical Center, Leiden, the Netherlands (E. van der Meijden, S. Kazem, M.M. Burgers, J.N. Bouwes Bavinck, M.C.W. Feltkamp);; Jeroen Bosch Ziekenhuis, ‘s Hertogenbosch, the Netherlands (R. Janssens);; National Institute of Public Health and the Environment, Bilthoven, the Netherlands (H. de Melker)

**Keywords:** trichodysplasia spinulosa, trichodysplasia spinulosa–associated virus, viruses, polyomavirus, immunoassay, seroprevalence, epidemiology, immunocompromised host, the Netherlands, research

## Abstract

We identified a new polyomavirus in skin lesions from a patient with trichodysplasia spinulosa (TS). Apart from TS being an extremely rare disease, little is known of its epidemiology. On the basis of knowledge regarding other polyomaviruses, we anticipated that infections with trichodysplasia spinulosa–associated polyomavirus (TSV) occur frequently and become symptomatic only in immunocompromised patients. To investigate this hypothesis, we developed and used a Luminex-based TSV viral protein 1 immunoassay, excluded cross-reactivity with phylogenetically related Merkel cell polyomavirus, and measured TSV seroreactivity. Highest reactivity was found in a TS patient. In 528 healthy persons in the Netherlands, a wide range of seroreactivities was measured and resulted in an overall TSV seroprevalence of 70% (range 10% in small children to 80% in adults). In 80 renal transplant patients, seroprevalence was 89%. Infection with the new TSV polyomavirus is common and occurs primarily at a young age.

Trichodysplasia spinulosa (TS) is a rare disease of the skin seen in solid organ transplant patients receiving immunosuppressive therapy (*1**–**5*) and in lymphocytic leukemia patients (*4**,**6**–**8*). A total of 15 TS cases have been described, of which 3 were identified in 2010 (*9**–**11*). The disease is characterized by development of follicular papules and keratin spines (spicules) predominantly in the face, often accompanied by alopecia of the eyebrows and eyelashes. Histologically, TS is characterized by abnormal maturation and marked distention of hair follicles. The inner root sheath cells are highly proliferative and contain excessive amount of trichohyalin (*1*). Transmission electron microscopy showed virus particles 40–45 nm in diameter within these cells (*1**,**4**,**6*).

In plucked spicules of a TS patient, we recently identified a new human polyomavirus virus known as TS-associated polyomavirus (TSV) (*9*). This finding has been recently confirmed by Matthews et al. (*12*). Recent analyses by our group have shown high copy numbers of TSV in lesions from other TS patients (S. Kazem and M.C.W. Feltkamp, unpub. data), underscoring the concept that TSV is the causative infectious agent. Phylogenetic analysis showed that TSV forms a tight cluster with a Bornean orangutan polyomavirus and among human polyomaviruses is most closely related to Merkel cell polyomavirus (MCV; also known as MCPyV) (*9*).

Eight human polyomaviruses have been identified: BKV (*13*), JCV (*14*), KIV (*15*), WUV (*16*), MCPyV (*17*), human polyomavirus type 6 (HPyV6) and type 7 (HPyV7) (*18*), and TSV (*9*). Infections with BKV and JCV are common and occur primarily in childhood without symptoms, after which the person remains persistently infected. Reactivation occurs only in immunocompromised patients and can cause serious disease, such as BKV-associated nephropathy and progressive multifocal leukoencephalopathy, and probably TS.

In immunocompetent populations, high seroprevalence values of 82%–98% for BKV (*19**–**22*) and 39%–77% for JCV (*19**–**22*) have been reported. For KIV and WUV identified in airway specimens, calculated seroprevalences are high in the general population (55%–90% and 69%–98%, respectively) (*20**,**21**,**23**,**24*). For MCPyV, which is present in ≈80% of rare but aggressive cutaneous Merkel cell carcinomas (MCCs) (*17**,**25**–**27*), seroprevalence among healthy persons was shown to be 42%–77% (*20**,**21**,**28**,**29*). A recent study reported higher serologic responses in MCC patients than in healthy controls (*30*).

Seroepidemiologic data for BKV, JCV, and MCPyV indicate that human polyomavirus infections are ubiquitous and generally occur without apparent disease. TSV seems to fit this profile, but no seroepidemiologic data to confirm this hypothesis are available. We report development and performance of a multiplex immunoassay to measure seroreactivity against TSV in immunocompetent persons, immunosuppressed persons, and a TS patient. Seroprevalences of TSV infection were calculated for persons of different ages and immune status. We show that TSV is a common infection in the general population and in immunocompromised patients, and discuss the relevance of our findings with respect to TSV-induced disease.

## Materials and Methods

### Generation of pGEX-TSV VP1 Expression Plasmid and GST-VP1 Fusion Protein Expression

To express TSV viral protein 1 (VP1) as a glutathione-S-transferase (GST) fusion protein, we created a pGEX4t3-TSV VP1.tag plasmid. For cloning of TSV VP1, sense (5′-GGATCCGGATCCGCCCCCAAAAGAAAAGG-3′) and antisense (5′-GTCGACGTCGACATAAAGCCGGGCGGGGAAG-3′) primers (*Bam*HI and *Sal*I restriction sites are underlined) were generated (Eurogentec, Cologne, Germany). Using these primers, we performed a PCR on the pUC19-TSV plasmid (*9*). A 2-step AmpliTaq gold PCR program was performed as described (*9*). TOPO TA cloning (Invitrogen, Carlsbad, CA, USA) of the amplified PCR product resulted in a construct used for cloning TSV VP1 into the pGEX4t3-BKV VP1.tag plasmid (*18*) after removal of the BKV VP1 sequence. The pGEX4t3-TSV VP1.tag construct was verified by sequencing using the BigDye Terminator Kit (Applied Biosystems, Foster City, CA, USA) and analyzed on an ABI Prism 3130 Genetic Analyzer (Applied Biosystems).

We sequenced VP1 from MCV isolate 344 and verified amino acid residues aspartic acid (D) and arginine (R) at positions 288 and 316 as found in MCV isolates 339 and 162. These residues are likely involved in proper folding of the VP1 for conformation-dependent epitope recognition (*21*). A pGEX4t3-tag plasmid was included to express tagged GST alone, which is necessary for serologic background determinations. In every construct, the tag sequence included codes for the 11-aa KPPTPPPEPET epitope of simian virus 40 (SV40) large T-antigen (*31**,**32*). GST and GST-fusion proteins of TSV, BKV, and MCPyV VP1 were expressed in the Bl21 Rosetta *Escherichia coli* strain as described (*21**,**31**–**33*). Expression of the GST-fusion proteins was analyzed by using Western blotting.

### Samples

A total of 528 serum samples from a population-based serum bank of healthy persons from the Province of Utrecht in the Netherlands were analyzed. This serum bank was set up in 1994 as a pilot study, the prePienter study, for a nationwide serum bank that would be used to evaluate long-term seroepidemiologic changes of diseases included in the Dutch National Immunization Program (*34*) (M.A. Conyn van Spaendonk et al., pilot study for Pienter project, logistical evaluation (part 1), RIVM-report no. 213675001/1995). Approval of the prePienter study was obtained from the Medical Ethical Committee of the Dutch Organization for Applied Scientific Research (TNO) (Leiden, the Netherlands), and every participant provided written informed consent. The age distribution within the population was <1–9 years, n = 79; 10–19 years, n = 66; 20–29 years, n = 51; 30–39 years, n = 64; 40–49 years, n = 76; 50–59 years, n = 54; 60–69 years, n = 79; and 70–79 years, n = 56.

We also tested 80 serum samples obtained in 1995 from immunocompromised renal transplant patients who came to a specialized dermatologic outpatient clinic at Leiden University Medical Center. These samples were obtained after informed oral consent was obtained from the patients, which was documented in patient files. The Medical Ethics Committee of Leiden University Medical Center reviewed and approved this study. The average age of the patients was 45 years (range 26–64 years).

A serum sample was also obtained from a 16-year-old immunocompromised heart transplant patient with TS. A detailed description of this patient was reported by van der Meijden et al. (*9*). The TS patient and his mother provided oral informed consent for the patient to provide serum for detection of antibodies against TSV, which was recorded in the patient’s medical file. The Medical Ethics Committee of the Leiden University Medical Center declared in writing that no formal ethical approval was needed to analyze this sample for viral diagnosis.

### Multiplex Serologic Analysis

Samples were analyzed for polyomavirus seroreactivity by using the multiplex antibody-binding assay developed and described by Waterboer et al. (*33*). Briefly, glutathione–casein (GC) coupled Bio-Plex polystyrene beads (Bio-Rad Laboratories, Hercules, CA, USA) containing a combination of fluorescent dyes were coupled to either GST-TSV VP1.tag, GST-BKV VP1.tag, GST-MCV VP1.tag, or GST.tag. For each antigen, 3,000 GC-coupled beads per sample were loaded with crude bacterial lysates containing relevant GST fusion protein. Samples were preincubated with GST.tag containing bacterial crude lysates (2 mg/mL) in blocking buffer to reduce nonspecific GST binding. For cross-reactivity studies, samples were preincubated with GST-TSV VP1.tag, GST-MCV VP1.tag, or GST-BKV VP1.tag. After preincubation, antigen-coated bead mixtures were incubated with samples diluted 1:100. For detection of bound serum antibodies, beads were incubated with goat anti-human total immunoglobulin G–biotin (1:1,000 dilution; Jackson ImmunoResearch Laboratories Inc., West Grove, PA, USA), streptavidin R–phycoerythrin (1:1,000 dilution; Invitrogen), and washed. Beads were analyzed in a Bio-Plex 100 analyzer (Bio-Rad Laboratories). Results are presented as median fluorescent intensity (MFI) units. For each sample, antigen-specific binding was obtained by subtracting the MFI for beads coated with GST alone from those of beads coated with GST VP1.

## Results

### Development of the TSV VP1 Immunoassay

**Figure 1:**
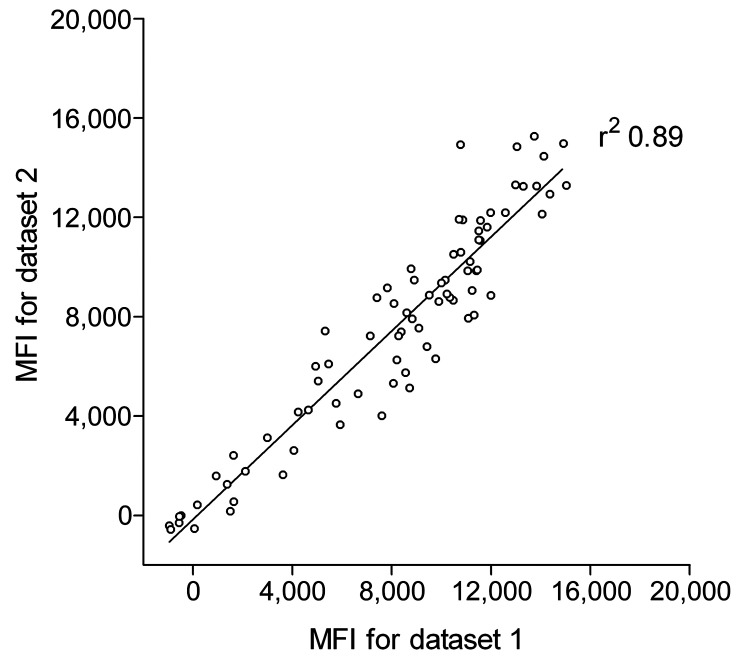
Reproducibility of trichodysplasia spinulosa–polyomavirus (TSV) viral protein 1 (VP1) immunoassay. Seroreactivity against TSV VP1 in 80 renal transplant patients, the Netherlands, was analyzed twice by using the Bio-Plex 100 analyzer (Bio-Rad Laboratories, Hercules, CA, USA). Datasets 1 and 2 were obtained during a 3-month interval by using freshly coupled identical glutathione–casein bead sets coupled independently with the same crude TSV VP1 bacterial extract. Each circle represents 1 serum sample, and the line represents results of linear regression analyses. Correlation coefficient (r^2^) was determined by using GraphPad Prism software (GraphPad Software Inc., La Jolla, CA, USA). MFI, median fluorescent intensity.

To measure seroreactivity against TSV, an immunoassay was developed with TSV VP1 antigen expressed as a GST-fusion protein in *E*. *coli*. The TSV VP1 immunoassay was developed according to the Luminex-based assay described by Waterboer et al. for simultaneous measurement of seroresponses against different human papillomavirus types (*33*). We first analyzed the reproducibility of the new assay with 80 serum samples from renal transplant patients. These samples were tested 3 months apart by using GC-coated beads coupled independently to the same crude TSV VP1 bacterial extract. This comparison showed reproducible results with a correlation coefficient of r^2^ 0.89 (Figure 1).

**Figure 2:**
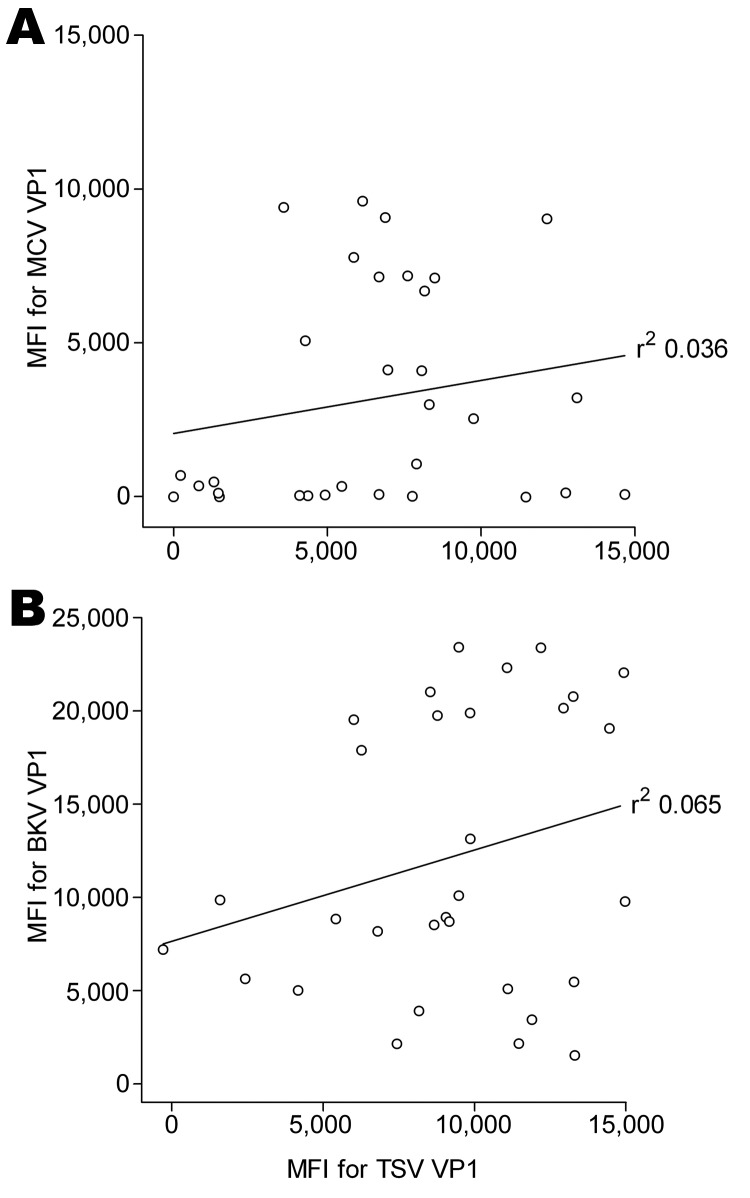
Cross-reactivity between trichodysplasia spinulosa–polyomavirus (TSV), Merkel cell polyomavirus (MCV), and BKV polyomavirus viral protein 1 (VP1). Correlation between seroreactivity against TSV VP1 and MCV VP1 (A) and BKV VP1 (B) was analyzed by using Bio-Plex 100 analyzer (Bio-Rad Laboratories, Hercules, CA, USA) with 30 serum samples from renal transplant patients, the Netherlands. Each circle represents 1 serum sample, and the line represents results of linear regression analyses. Correlation coefficients (r^2^) were determined by using GraphPad Prism software (GraphPad Software Inc., La Jolla, CA, USA). MFI, median fluorescent intensity.

Although not expected on the basis of amino acid sequence comparison (RefSeq TSV NC_014361 and MCV: NC_010277) for a randomly selected subset of 30 renal transplant serum samples, we investigated a possible association between seroreactivity against VP1 of TSV and that of MCPyV because MCPyV is phylogenetically the closest related human polyomavirus to TSV (*9*). No association between TSV and MCPyV VP1 seroresponses was observed (r^2^ 0.036; Figure 2, panel A). Similar findings were obtained when TSV VP1 seroresponses were compared with those against the more distantly related BKV VP1 (r^2^ 0.065; Figure 2, panel B).

**Figure 3:**
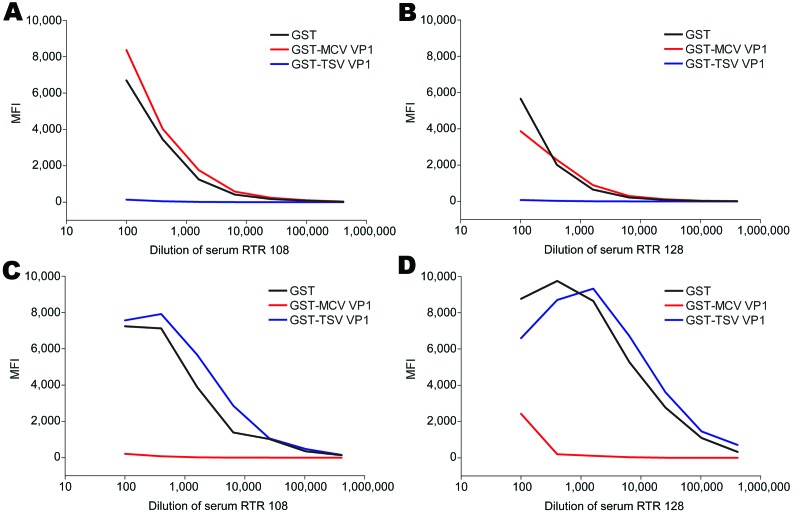
Cross-competition between trichodysplasia spinulosa–associated polyomavirus (TSV) and Merkel cell polyomavirus (MCV) viral protein 1 (VP1) in serial dilutions of serum samples RTR 108 and RTR 128 from renal transplant recipient patients reactive against TSV VP1 and MCV VP1, the Netherlands. Reactivity was determined by using the VP1 multiplex antibody-binding assay. Samples were preincubated with soluble recombinant glutathione-S-transferase (GST) (black line), GST-MCV VP1 (red line), or GST-TSV VP1 (blue line). Values are median fluorescent intensity (MFI) for seroreactivity against TSV VP1 (A and B) or MCV VP1 (C and D).

**Figure 4:**
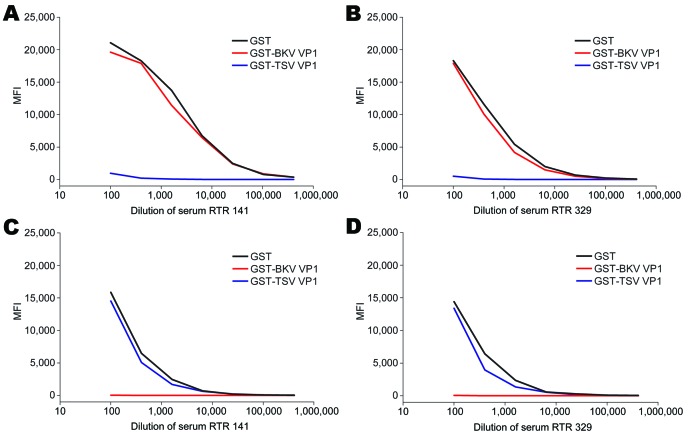
Cross-competition between trichodysplasia spinulosa–associated polyomavirus (TSV) and BKV polyomavirus viral protein 1 (VP1) in serial dilutions of serum samples RTR 141 and RTR 329 from renal transplant recipient patients reactive against TSV VP1 and BKV VP1, the Netherlands. Reactivity was determined by using the VP1 multiplex antibody-binding assay. Samples were preincubated with soluble recombinant glutathione-S-transferase (GST) (black line), GST-BKV VP1 (red line), or GST-TSV VP1 (blue line). Values are median fluorescent intensity (MFI) for seroreactivity against TSV VP1 (A and B) or BKV VP1 (C and D).

We also evaluated potential cross-reactivity between TSV VP1 and MCPyV VP1 in detail for 2 TSV- and MCPyV-reactive serum samples. These samples were titrated and preincubated with soluble GST, GST-TSV VP1, or GST-MCV VP1. Subsequently, TSV VP1 and MCPyV VP1 seroresponses were measured. In both samples tested, TSV VP1 reactivity was inhibited by preincubation with TSV VP1 only and not with MCPyV VP1, whereas MCPyV VP1 reactivity could be inhibited by preincubation with MCPyV VP1 only (Figure 3). Similar results were obtained in a TSV VP1 and BKV VP1 competition experiment with TSV-reactive and BKV-reactive samples (Figure 4).

### TSV VP1 Seroresponse in a TS Patient

TSV seroreactivity was determined in a TS patient previously reported (*9*). A serum sample was obtained 6 months after detection of TSV and daily facial treatment with cidofovir-containing cream had started. At the time the sample was obtained, treated lesions had resolved but untreated skin (e.g., of the legs) still had typical spicules indicative of active TSV infection.

**Figure 5:**
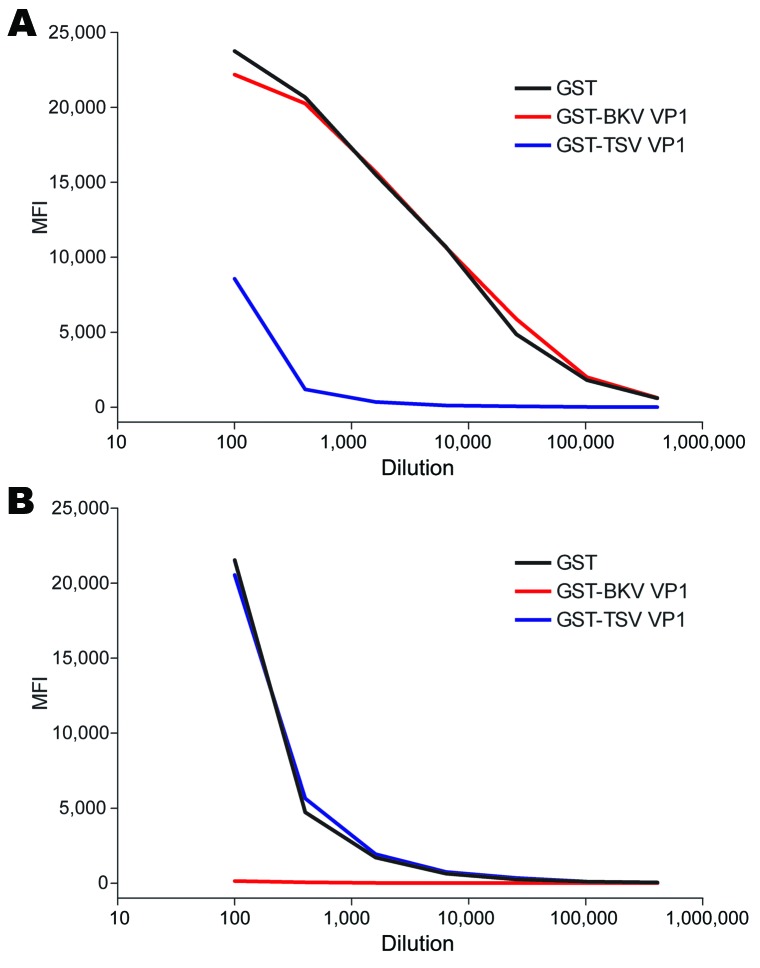
Seroresponses against trichodysplasia spinulosa–associated polyomavirus (TSV) (A) and BKV polyomavirus (B) for a patient with trichodysplasia spinulosa, the Netherlands. Serial dilutions of serum from a TS patient were tested for reactivity against TSV viral protein 1 (VP1) or BKV VP1 by using the VP1 multiplex antibody-binding assay. Samples were preincubated with soluble recombinant glutathione-S-transferase (GST) (black line), GST-BKV VP1 (red line), or GST-TSV VP1 (blue line). MFI, median fluorescent intensity.

Serial dilutions of the TS serum sample were tested by using the TSV VP1 assay. High reactivities were observed (Figure 5, panel A). This response could be exceeded by using soluble GST-TSV VP1 but not with GST-BKV VP1. Conversely, the BKV seroresponse observed in this patient was exceeded only by using soluble recombinant GST-BKV VP1 and not by using GST-TSV VP1 (Figure 5, panel B). No seroresponse against MCV VP1 was detected for this patient.

### TSV Seroresponses in Healthy and Immunocompromised Populations

**Figure 6:**
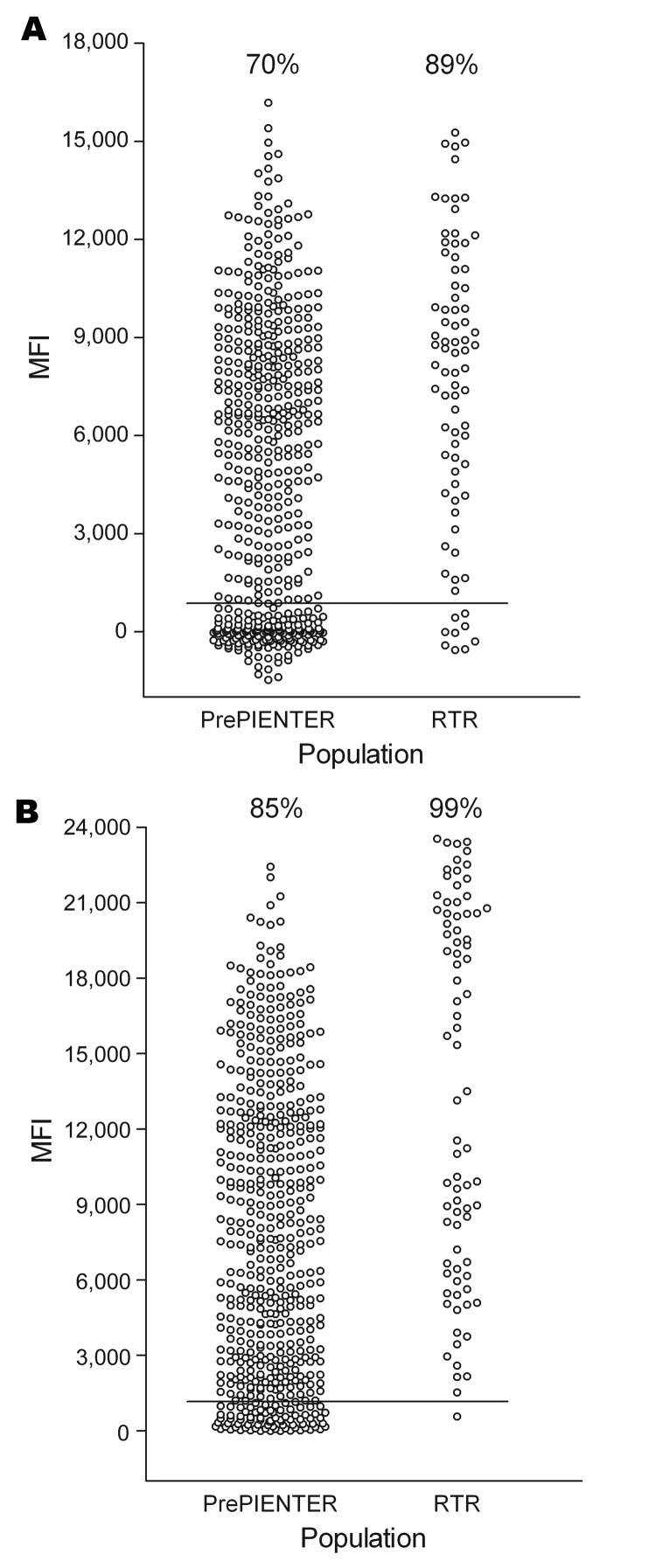
Seroresponses to trichodysplasia spinulosa–associated polyomavirus (TSV) and BKV polyomavirus in healthy and immunocompromised populations, the Netherlands. Serum samples were obtained from 528 healthy persons (PrePIENTER) and 80 renal transplant recipients (RTR) and screened for reactivity against TSV viral protein 1 (VP1) (A) and BKV VP1 (B) by using the VP1 multiplex antibody-binding assay. Each circle represents 1 sample, and horizontal lines represent cutoff values. Percentage values indicate seropositivity. MFI, median fluorescent intensity.

TSV VP1 seroreactivity was determined for 528 healthy persons and 80 immunosuppressed renal transplant patients. BKV VP1 was included in the analyses as a positive control because of the known high BKV seroprevalence in the general population (*19**–**21*). In every experiment, a panel of 3 reference serum pools was also included, which showed little variance over time. Results for TSV VP1 and BKV VP1 are shown in Figure 6.

**Figure 7:**
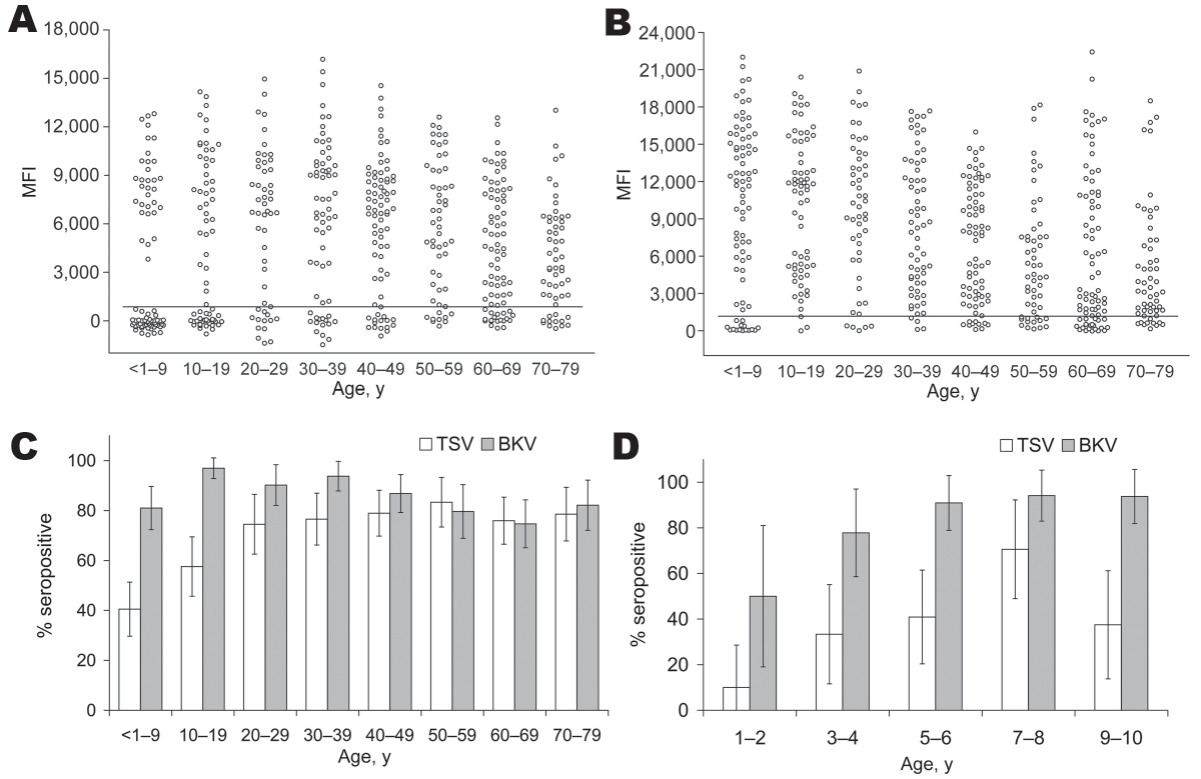
Age-related seroprevalence of trichodysplasia spinulosa–associated polyomavirus (TSV) viral protein 1 (VP1) (A) and BKV polyomavirus VP1 (B) in a healthy population, the Netherlands. The population was divided into 8 age groups: <1–9 years of age (n = 79), 10–19 (n = 66), 20–29 (n = 51), 30–39 (n = 64), 40–49 (n = 76), 50–59 (n = 54), 60–69 (n = 79), and 70–79 (n = 56). Each circle represents 1 serum sample, and the horizontal lines represent cutoff values. MFI, median fluorescent intensity. C) Seroprevalence of TSV VP1 (white bars) and BKV VP1 (gray bars), by age. D) Seroprevalence of TSV VP1 and BKV VP1 in youngest age group. Population was divided into 5 smaller age groups: 1–2 years of age (n = 10); 3–4 (n = 18); 5–6 (n = 22); 7–8 (n = 17); 9–10 (n = 16). Error bars indicate 95% confidence intervals.

To investigate age-specific TSV seroreactivity and to calculate a cutoff value to determine TSV seropositivity, we subdivided the healthy population into different age groups (Figure 7, panel A). For persons <1–9 years of age, a clear distinction could be made between patients who were seronegative for TSV (MFI ≈0) and children with TSV seroreactivities of 4,000–12,000 MFI units. To calculate TSV seropositivity, a cutoff value of 877 MFI units was calculated on the basis of mean seroreactivity of the TSV serononresponders from the lowest age group + 3 SD. Although the distinction between seronegative persons and seropositive persons in the first age group was less clear for BKV (Figure 7, panel B), a similar strategy was used for BKV and resulted in a cutoff value of 1,051 MFI units.

### TSV Seroprevalence in Healthy and Immunocompromised Populations

Using the calculated cutoff values, we determined the age-specific seroprevalence for TSV in each age group of the immunocompetent population. In the children <10 years of age, the seroprevalence for TSV was 41% (Figure 7, panel C). This percentage increased to ≈75% at 30 years of age, and remained stable for higher age groups. BKV seroprevalences were calculated and showed values between 75% and 97% (Figure 7, panel C). Analyses of the youngest age group showed an increasing trend for TSV and BKV seropositivity starting at 10% for TSV in children 1–2 years of age (Figure 7, panel D).

On the basis of these calculations, overall seroprevalence for TSV VP1 was 70% for the healthy population and 89% for the immunocompromised population (Figure 6, panel A). For BKV VP1, the overall seroprevalence for both groups was somewhat higher (85% and 99%, respectively) (Figure 6, panel B).

## Discussion

To investigate the seroepidemiologic aspects of TSV infection, we developed a multiplex immunoassay. This approach was based on Luminex technology and shown to be a reliable method for seroepidemiologic studies of papillomavirus and polyomavirus infections (*19**,**21**,**33**,**35**,**36*). The choice for VP1 as antigen of interest was governed by results of studies on BKV, JCV, and SV40 polyomavirus, which showed that the major capsid protein is immunodominant (*19*). However, the less immunogenic large T-antigen may also be useful in discriminating active TSV infections from latent infections because it has been reported that antibodies against MCPyV T antigens reflect the tumor incidence for MCC patients (*37*).

The TSV VP1 immunoassay was reproducible and showed minimal signs of TSV cross-reactivity with MCPyV. Cross-reactivity studies have shown correlations between serorecognition of SV40 and BKV only, and to a lesser extend between SV40 and JCV (*20**,**38**,**39*), all of which are more closely related than TSV and MCPyV (*9*). Detailed comparison of antigenic VP1 loop regions of TSV, MCPyV, and BKV, as performed for KIV, WUV, MCPyV, and lymphotropic polyomavirus by Kean et al. (*20*), also showed little similarity. On the basis of the new polyomavirus phylogenetic tree that was recently published (*9*), only cross-reactivity between TSV and the closely related Bornean orangutan polyomavirus 1 might have been expected. However, this animal virus was not included in this human study.

Seroreactivity of the symptomatic TS patient was the highest of all participants in the study. Even at a dilution of 1:100,000, some reactivity above background was detected, which indicates a high concentration of TSV-specific antibodies in this patient. This interpretation was also suggested by the observation that at the highest serum concentration, competition with soluble GST-TSV VP1 did not result in complete inhibition of TSV seroreactivity. This finding might be unexpected because immunosuppressed patients are often considered less immunoreactive. However, the immunosuppressive regimens are aimed to decrease cellular immunity to prevent donor organ rejection. It is anticipated that polyomavirus-specific cellular immunity will be decreased by such a regimen, which would increase the pool of infected cells and produce larger amounts of virus, even viremia. As a result, memory B cells may become activated and production of TSV-specific antibodies will increase accordingly.

The seroprevalence we calculated for TSV among the healthy population was high and comparable with that found for other human polyomaviruses (*20**–**24**,**28**,**29*). Therefore, TSV seems to be a ubiquitous virus that frequently causes infection in the general human population. A total of 41% of the children <1–9 years of age were seroreactive to TSV and therefore likely infected. Whether TSV infections persist is not known, but this persistence is likely on the basis of results for other polyomavirus infections.

The calculated overall TSV seroprevalence was higher for the immunocompromised group than for healthy persons. When age was taken into account, we observed that the difference in TSV prevalence between both populations was of borderline significance (p = 0.03). As discussed for the TS patient, this seemingly paradoxical phenomenon might be explained by increased humoral immunity against TSV as a result of increased viral activity under (cellular) immunosuppression. Whether this hypothesis involves TSV reactivations or reinfections is not known. However, it is also not known whether overt TS reflects a fulminant primary TSV infection or a symptomatic reactivation.

In conclusion, by using a newly developed immunoassay, we were able to measure TSV seroreactivity with high reproducibility and low cross-reactivity. We calculated the seroprevalence of TSV in healthy persons and provided evidence that TSV is a common circulating virus in the general population in the Netherlands that preferentially infects persons at an early age. Additional studies will need to determine whether TSV infections remain persistent in the host, as shown for other polyomaviruses, and what triggers TSV reactivation. The fact that symptomatic TS is such a rare condition suggests that there are more factors involved in this condition than immunosuppression alone.
